# A Critical Review of the Evidence Concerning the HIV Latency Reversing Effect of Disulfiram, the Possible Explanations for Its Inability to Reduce the Size of the Latent Reservoir In Vivo, and the Caveats Associated with Its Use in Practice

**DOI:** 10.1155/2017/8239428

**Published:** 2017-03-30

**Authors:** Harry D. J. Knights

**Affiliations:** Brasenose College, University of Oxford, Oxford, UK

## Abstract

Combination antiretroviral therapy (cART) effectively suppresses the replication of human immunodeficiency virus type 1 (HIV-1), improves immune function, and decreases the morbidity of acquired immune deficiency syndrome (AIDS). However, it is unable to eradicate the virus because it does not eliminate latently infected cells. The latent reservoir poses the major barrier to an HIV-1 cure. The “shock and kill” strategy aims to reactivate the virus and destroy latently infected cells. Many latency reversing agents (LRAs) reactivate HIV in vitro, but the absence of damaging side-effects and efficacy in vivo make disulfiram particularly promising. However, in clinical trials to date, disulfiram treatment has not resulted in a reduction in the size of the latent reservoir. In this article I will therefore discuss the evidence for the latency reversing effect of disulfiram, the possible explanations for its inability to reduce the size of the latent reservoir in vivo, and the caveats associated with its use in practice. These considerations will help to inform judgements about the prospect of an HIV cure from disulfiram based treatments.

## 1. Introduction

Combination antiretroviral therapy (cART) for people infected with human immunodeficiency virus type 1 (HIV-1) effectively suppresses viral replication to below the limit of detection [1]. This successfully improves immune function and substantially decreases the morbidity and mortality of acquired immune deficiency syndrome (AIDS) [1]. However, it fails to eliminate the virus because a latent form of HIV persists [2, 3].

The latent reservoir consists of between 10^5^ and 10^6^ long-lived resting memory CD4^+^ T cells (Tm) harbouring an integrated, replication competent HIV genome [2–4]. Provided the host cell stays quiescent, the provirus remains transcriptionally silent [5]. This means that latently infected cells do not express viral antigen and therefore cannot be targeted by the immune system [3, 5]. However, following cellular activation, the provirus is transcribed and virus is produced, reestablishing active infection [2, 3]. The latent reservoir is extremely stable; it has been estimated to have a half-life of 44 months in HIV patients receiving cART, requiring 73.4 years to be completely destroyed [6]. This might be explained by the vertical transmission of infectious proviruses during the clonal expansion of Tm cells [7]. The recently discovered long-lived latently infected stem cell-like Tm cells (Tscm) [8] may also be involved. However, the significance of this rare population of cells remains unclear.

The stability of the latent reservoir means that HIV patients have to maintain cART for life. This is expensive, requires lifelong compliance, has undesirable side-effects, and creates stigma, providing the rational for the development of an HIV cure. Two types of cure are recognised: sterilising and functional. A sterilising cure would completely eradicate the virus (including latent virus), while a functional cure would result in long-term host-mediated control of viral replication and consequently lasting remission of symptoms in the absence of cART, with replication competent virus remaining within the body.

The “shock and kill” [9] strategy has recently gained a lot of attention. This aims to reactivate the latent virus, reducing the size of the latent reservoir through direct viral cytopathic killing and immune-mediated destruction while cellular reinfection is blocked with cART [9]. Although this has traditionally been considered a sterilising cure strategy, recent studies on the natural hosts of Simian immunodeficiency viruses (SIVs) suggest that reduced central memory CD4^+^ T cell (Tcm) infection, in the absence of viral eradication, might be responsible for their functional cure [10, 11]. It is therefore tempting to speculate that incomplete shock and kill strategies might reduce the size of the Tm pool enough for continued antiretroviral treatment to establish a functional cure in humans. Therefore, precisely what type of cure shock and kill is aiming to achieve is open to debate.

Early shock and kill attempts revolved around the use of immune activators: anti-CD3 immunoglobulin, IL-2, and TNF*α* [12, 13]. However, they entailed a high risk of global T cell activation and systemic induction of proinflammatory cytokines [12, 13]. Therefore, latency reversing agents (LRAs) that activate HIV from latency without global T cell activation were needed. Since the turn of the century, an array of different compounds have shown promise in vitro [14–16]. Two of these, the histone deacetylase inhibitors (HDACI) [17–19] and disulfiram [20, 21], have recently shown success in vivo. However, there have been indications in the literature of widespread and damaging HDACI side-effects [22], including disruption of the antiviral immune system [23], raising concerns about the feasibility of prolonged administration. Accordingly, in this article I will focus on disulfiram, although other LRAs will be discussed where appropriate.

### 1.1. Disulfiram

**Figure 1:**
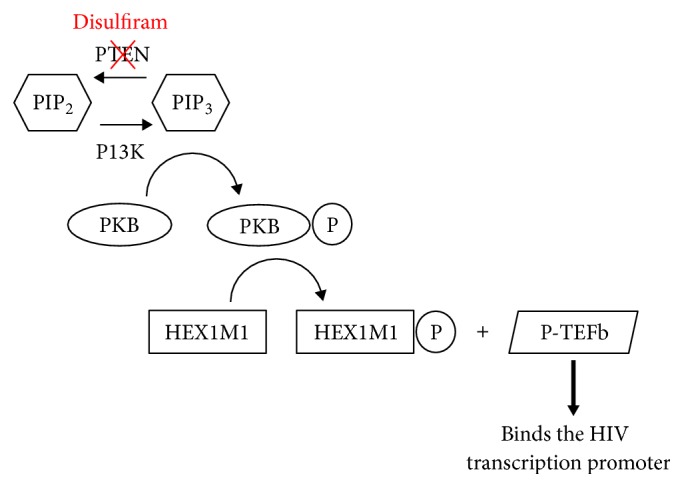
The mechanism of disulfiram activation of HIV transcription. Disulfiram activates the PKB/Akt signalling pathway by depleting PTEN (see Doyon et al. [25]).

The latency reversal capabilities of disulfiram and its metabolites were first uncovered in 2011 by Xing et al. [15]. They established latency in CD4^+^ lymphoblasts by infecting them with HIV and then allowing them to return to a resting state. Elegantly, since they used recombinant HIV expressing GFP, they were able to assess reactivation by flow cytometry. Using this technique, they screened the John Hopkins Drug Library and found eight hits; one of them, disulfiram, had been used to safely treat alcoholism for decades under the trade-name “Antabuse” [24]. Crucially, disulfiram treatment did not increase cell size (an indicator of activity), the production of proinflammatory cytokines, or the expression of activation markers. Therefore, the latency reversal effect of disulfiram appeared to be HIV specific and not due to global T cell activation. The proposed mechanism is outlined in Figure 1.

The in vitro effect, however, did not translate into an effect on patient cells. In 2014, Bullen et al. examined the latency reversal effect of disulfiram on resting CD4^+^ T cells freshly isolated from HIV patients on cART [26]. Unexpectedly, they found that disulfiram did not induce viral outgrowth and virus release or cause increases in intracellular HIV mRNA. This suggested, in contrast to studies on in vitro models, that disulfiram does not reactivate latency in patient cells. The reasons for this discrepancy are unclear. One explanation is that current in vitro models for HIV latency do not fully recapitulate the mechanisms governing HIV latency in patient cells. Disappointingly, therefore, this study suggested that disulfiram would be unlikely to drive eradication of the latent reservoir in vivo.

At first, this was consistent with evidence from clinical trials. In 2014, Spivak et al. [20] administered 500 mg of disulfiram daily for two weeks to HIV patients on stable cART. While cART is extremely effective at suppressing viral replication, patients on cART still develop a residual viraemia [27–29]. This is caused by the release of virus from latently infected cells following activation [28]. Therefore, the authors were able to determine latency reactivation in vivo by measuring plasma HIV RNA. In the original analysis, they found no significant change to plasma HIV RNA during disulfiram administration. However, in a post hoc analysis, a transient increase was detected in patients sampled immediately after dosing and patients with higher plasma disulfiram concentrations. These findings inspired a subsequent phase 2 dose-escalation study [21]. Patients received 500, 1000, or 2000 mg of disulfiram daily for three days. A dose-related increase in cell-associated unspliced HIV RNA was observed during and after dosing. It remains unclear why Bullen et al. were unable to detect the same increase in intracellular HIV RNA ex vivo on patient cells. It is possible the immune system is having a synergistic effect on latency reactivation in vivo [26]. Collectively, these landmark clinical studies suggest that disulfiram provides a window of effective latency reactivation in vivo. This provides the first evidence of successful latency reversal in HIV infected individuals by a safe, well-tolerated drug and well-established drug.

## 2. Discussion

There are a number of problems with the use of disulfiram to eradicate the latent reservoir that need to be discussed.

### 2.1. The Persistence of the Latent Reservoir

Inconsistent with the shock and kill hypothesis, none of the disulfiram studies reported a decrease in the frequency of latently infected cells. Understanding why this is the case will be essential for the development of effective shock and kill cure strategies.

Disulfiram might simply not be potent enough to achieve the magnitude of latency reversal required to cause virus-mediated cell lysis. If correct, it might be necessary to use disulfiram in combination with other mechanistically distinct LRAs to enhance the reactivating effect [30]. This might be compounded by the high frequency of viral escape mutations in patients on cART [31] and the impaired cytotoxic T cell (CTL) cytolytic capacity associated with chronic HIV infections [32], both predicted to inhibit the immune-mediated destruction of reactivated cells.

Interestingly, this is consistent with a study by Shan et al. showing that in vitro latency reversal using an HDACI, suberoylanilide hydroxamic acid (SAHA), did not result in the death of infected Tm cells despite viral cytopathic effects and the presence of autologous CTLs [33]. Instead, the efficient killing of infected cells required antigen-specific stimulation of patient CTLs prior to latency reactivation. It therefore appears that a balance between overstimulation, leading to global T cell activation, and understimulation, leading to the survival of reactivated cells, needs to be achieved. This has led to the general consensus that prestimulation of the antiviral immune response might be necessary for the successful “kill” of reactivated cells. Further studies will be required to confirm whether this applies to disulfiram in vivo.

### 2.2. The Presence of Other Viral Reservoirs

Many studies have examined the effect of disulfiram on latently infected Tm cells. However, less have investigated the effect of disulfiram on other cells. Many different cell types including macrophages [34], dendritic cells [35], and haematopoietic progenitor cells (HPCs) [36] are susceptible to HIV in vitro. Moreover, macrophages [34], microglia [37, 38], astrocytes [37–39], and HPCs [36] from HIV patients have been shown to contain HIV DNA. These findings have led to the proposition that latently infected Tm cells might not be the only viral reservoir in vivo. Whether or not this is the case is still far from certain. While other cell types can be infected in vivo and contain integrated HIV genomes, whether they persist for years in the setting of cART and can be reactivated to produce infectious virus is a contentious issue [40] (for a review, see Kandathil et al. 2016 [41]). Therefore, from a clinical perspective, it is unclear whether they are capable of reseeding infection.

Crucially, however, if non-Tm reservoirs do develop, they will be important to eradication efforts and will likely become more so once the Tm reservoir has been eliminated. Specifically, if disulfiram cannot target these reservoirs, cART could never be stopped for fear of viral rebound “restocking” the cleared reservoirs.

### 2.3. The Possibility of a CNS Reservoir

Recently, the possibility of a CNS reservoir has been the centre of attention [40]. This is because the CNS has a unique set of characteristics that could greatly affect the outcome of LRA use:

1. The potential to develop HIV-associated neurocognitive disorders (HANDs) [42].
2. Restricted LRA penetration [43] which may limit the “shock.”
3. Altered immune surveillance [44] which may compromise the “kill.”
4. Restricted cART penetration [45] which may allow continued viral replication, counteracting the clearance of the latent reservoir.
5. Phylogenetically distinct HIV clades within the CNS which may respond differently to the LRA [46].
6. Latently infected cells within the CNS (microglia and possibly astrocytes) are long-lived [47].

The development of HANDs is of particular importance. Owing to the success of cART, the life expectancy of HIV patients has increased dramatically over the last 20 years [48]. As a result, secondary HIV pathologies (such as HANDs) are becoming more frequent [40]. The pathophysiology underlying the development of HANDs is not known. However, one possibility is that they are caused by a long-lived infected CNS cell population that is releasing virus within the brains of suppressed patients. If this is the case, shock and kill strategies will need to target these cells. Conveniently, disulfiram easily crosses the blood-brain barrier because of its small size [49]. This means it is anatomically able to target any latently infected cells residing within the CNS. However, Gray et al. found it did not induce viral transcription in monocyte-derived macrophages, a cellular model for brain perivascular macrophages and microglia, or primary foetal astrocytes [50]. This result is disappointing: if reproducible in vivo, disulfiram may not improve the cognitive decline of HIV patients. Specific techniques for targeting CNS latency may therefore be needed in conjunction with disulfiram treatment.

However, the degree to which their cellular model represents latently infected CNS cells in vivo needs careful consideration. In particular, they validate their model by confirming the cell replication kinetics are equivalent to cultured microglia; this, however, is just one cell characteristic. They also used a lentiviral vector whose long-terminal repeat (LTR) was derived from the X4-tropic laboratory strain of HIV-1, NL4-3. The authors hypothesised that LTRs derived from CNS-tropic strains of HIV might respond differently to disulfiram. However, this is now known not to be the case. In a later study, the same group showed disulfiram did not activate transcription in human foetal astrocytic cells transfected with HIV containing LTRs from the CNS compartment of HIV patients [51]. Nevertheless, these findings need to be confirmed in animal models and then in vivo on HIV patients before any conclusions can be drawn about the efficacy of disulfiram on latently infected CNS cells.

On the other hand, if it transpires that disulfiram cannot target latently infected CNS cells, it is possible that this may not affect its therapeutic potential. Specifically, whether HANDs are caused by the release of virus from long-lived latently infected CNS cells remains unknown. Instead, it is possible that HANDs are a consequence of untreated viral infection prior to the initiation of cART or during periods of interrupted treatment. Moreover, only around 10% of HIV patients have detectable levels of HIV RNA in their cerebrospinal fluid (CSF) [52]. This might suggest that the presence of latently infected cells within the CNS is an uncommon occurrence. As a result, the precise implications of disulfiram being unable to reverse latency in CNS cells remain unclear.

Furthermore, it must also be considered that successful shock and kill within the CNS has the potential to cause cognitive decline itself through the destruction of infected glial cells and subsequent neuroinflammation. This would be a particular concern for patients with a larger number of latently infected cells within the CNS (perhaps because of late cART initiation or a long infection period). It might, therefore, be better to leave such patients on strict cART regimes. On the other hand, there may be situations where mild or transient neuronal dysfunction is an acceptable “price” for HIV eradication, similar to the “chemo brain” some patients experience following systemic cancer chemotherapy [53]. Either way, if LRAs like disulfiram were to be used clinically, neurocognitive monitoring would be essential to ensure any neurocognitive decline is detected in time for treatment to be stopped.

There is also the challenge of achieving adequate CNS penetration of antiretroviral agents to ensure the clearing of the CNS viral reservoir is not masked by continued CNS viral replication and propagation. The antiretroviral drug CNS penetration-effectiveness (CPE) rank [54] provides a useful guide and can be augmented by novel nanoformulation delivery techniques [55]. However, CNS penetration is potentially double-edged; in vitro studies suggest some antiretrovirals, particularly the NRTI abacavir and the NNRTI nevirapine, can be neurotoxic [56]. Therefore, while CNS cART infiltration will be essential for successful shock and kill, this problem should be approached with caution for fear of worsening any HANDs and impairing CNS function.

### 2.4. Measuring the Size of the Viral Reservoir

Parallel to the problem of eliminating the latent reservoir is the challenge of measuring the size of the latent pool to determine when the virus has been eradicated. This highlights another key problem facing efforts to eradicate HIV using disulfiram: techniques for measuring reservoir size are inaccurate and complicated. An in-depth analysis of the pros and cons of the different techniques used is beyond the scope of this article (for a review, see Bruner et al. [57]). However, in brief, there are two types: quantitative viral outgrowth assays (Q-VOA) and polymerase chain reaction- (PCR-) based methods.

The Q-VOA uses phytohemagglutinin to globally activate Tm cells ex vivo and reinstate HIV production. The virus is then expanded and quantified by ELISA for the HIV p24 antigen. While this approach has long been the “gold standard,” a recent study identified intact proviruses from p24 negative samples [58], suggesting that the Q-VOA underestimates the size of the in vivo reservoirs. This is believed to be because reactivation is stochastic, such that each provirus has a finite probability of being induced following activation [59]. Furthermore, the Q-VOA is labour intensive and expensive, has a 1–3 week culture time, and requires large blood volumes. Accordingly, it remains unsuitable for large scale clinical trials.

Alternatively, the PCR methods directly measure the frequency of cells containing HIV genomes [60]. Here, PCR is performed on blood Tm cell DNA and the infected cell frequency is calculated by comparison to a standard curve constructed using known copy numbers of proviral DNA. Although PCR methods are simple and require small blood volumes, they quantify large numbers of dysfunctional, replication incompetent viral genomes [61]. In fact, PCR methods typically yield infected cell frequencies 2-3 logs higher than the Q-VOA [61]. Generally, therefore, the Q-VOA provides an underestimate and PCR an overestimate, of the true size of the latent pool. Using this information, it would be useful to create an equation to more precisely calculate true reservoir size from the Q-VOA and PCR estimates. This could be done in vitro by controlling latent reservoir size, measuring it with the Q-VOA and PCR methods, and then comparing the values.

Furthermore, nonspecificity means it is very difficult for PCR methods to detect small changes in the number of intact proviruses capable of reseeding infection. This is problematic because very few latently infected cells contain an intact provirus [62], and therefore successful shock and kill will only result in a small absolute reduction in the number of intact proviruses. Therefore, it is possible that the disulfiram studies underestimated the latency reversing effect on replication competent provirus.

To this end, perhaps the best technique to date has been the* tat/rev *induced limiting dilution assay (TILDA) [63]. This measures the frequency of cells containing inducible multiply spliced* tat/rev *RNA. This is usually absent from latent cells and is only induced upon reactivation [64]. Conversely, many defective genomes have deletions encompassing the tat and rev genes [58]. As a result, the latent cell frequency measured by TILDA lies somewhere between the Q-VOA and PCR estimates, indicative of greater accuracy. There are also no RNA extraction or virus amplification steps, meaning it takes just two days, and it offers similar advantages to PCR with regard to simplicity and small blood volume requirement. This makes TILDA a potentially useful assay for large scale clinical trials. However, again there are limitations: it relies on the amplification of a highly variable HIV genome region, suggesting that the required primers may not recognise all viral quasispecies.

Furthermore, as previously discussed, other latent cell types exist within tissues. This would not be a hindrance if the size of blood and tissue reservoirs was tightly correlated, but there is conflicting and limited evidence for this [65]. It might therefore be necessary to combine these quantification methods with tissue sampling techniques. This has been done previously [66]. However, biopsies pose their own limitations: they are invasive and cannot be taken from all tissues, especially the CNS. For the CNS, it may be possible to estimate the number of latently infected cells from the CSF viral load. However, further studies will be needed to confirm whether CSF viral load is dependent on the frequency of latently infected cells that may be residing within the CNS.

As can be seen, all techniques for quantifying the reservoir have their own advantages and disadvantages. This poses a serious problem for HIV eradication efforts. Without a high-throughput, sensitive, and well-validated assay it will remain difficult for researchers to move forward with clinical trials, accurately evaluate the efficacy of LRAs like disulfiram, and ever confirm that a sterilising cure has been achieved.

## 3. Conclusions

Overall, there is strong evidence that disulfiram effectively and specifically reverses HIV latency in cellular models. While this failed to translate into an effect on patient cells, subsequent clinical trials identified an effect in vivo. However, so far, this has failed to result in a reduction in the size of the latent reservoir in HIV patients. Possible explanations for this include insufficient potency and failed immune clearance. Therefore, it is likely that combining disulfiram with other mechanistically distinct LRAs and the use of preimmune stimulation will help overcome these challenges. Furthermore, future studies will be needed to assess whether non-Tm reservoirs are able to reseed the infection after prolonged cART. If they are, safe shock and kill techniques for nonblood reservoirs will need to be explored. Finally, more refined techniques for measuring the size of the latent reservoir safely in different tissues need to be developed. Therefore, while disulfiram shows promise as an HIV cure strategy, further studies are needed to solve the problems highlighted in this article before any conclusions about its clinical efficacy can be drawn.
