# Corrigendum to “A Critical Review of the Evidence Concerning the HIV Latency Reversing Effect of Disulfiram, the Possible Explanations for Its Inability to Reduce the Size of the Latent Reservoir In Vivo, and the Caveats Associated with Its Use in Practice”

**DOI:** 10.1155/2019/4942573

**Published:** 2019-03-13

**Authors:** Harry D. J. Knights

**Affiliations:** Brasenose College, University of Oxford, Oxford, UK

In the article titled “A Critical Review of the Evidence Concerning the HIV Latency Reversing Effect of Disulfiram, the Possible Explanations for Its Inability to Reduce the Size of the Latent Reservoir In Vivo, and the Caveats Associated with Its Use in Practice” [1], the Acknowledgments should be revised as follows:

“The author acknowledges Professor William James for his assistance. The author gratefully acknowledges the Imperial College London library services for funding the open access article processing charges.”
